# Desarrollo y validación de Cefalytics: una aplicación basada en inteligencia artificial para el diagnóstico rápido de cefaleas primarias

**DOI:** 10.1016/j.aprim.2026.103530

**Published:** 2026-07-30

**Authors:** Josefina Martínez Simón, Pablo Arjona Ruiz, Jesús Martín Alcalá, Juan Manuel García-Torrecillas, María Fernández Recio, Antonio Arjona-Padillo

**Affiliations:** a Servicio de Neurología, Hospital Universitario Torrecárdenas, Almería, España; b Máster en Big Data; c Máster en Inteligencia Artificial; d Servicio de Urgencias y Unidad de Investigación Biomédica, Hospital Universitario Torrecárdenas, Almería; Centro de Investigación Biomédica en Red de Epidemiología y Salud Pública (CIBERESP), Madrid, España; e Servicio de Neurología, Hospital Virgen del Valme, Sevilla, España

**Keywords:** Cefalea primaria, Diagnóstico, Inteligencia artificial, Aprendizaje automático, Migraña, Primary Headache, Diagnosis, Artificial Intelligence, Machine Learning, Migraine

## Abstract

**Objetivo:**

Desarrollar y validar una aplicación en castellano basada en inteligencia artificial para apoyar el diagnóstico de cefaleas primarias por médicos no expertos.

**Diseño:**

Estudio observacional retrospectivo de desarrollo y validación diagnóstica basado en registros clínicos.

**Emplazamiento:**

Consultas de neurología que atienden pacientes derivados desde atención primaria (nivel asistencial 2 en cefaleas).

**Participantes:**

Se incluyeron 1.029 pacientes adultos con diagnóstico de 17 tipos de cefalea primaria confirmado por un neurólogo experto según los criterios de la tercera edición de la Clasificación Internacional de Cefaleas (ICHD-3), atendidos en 2 centros hospitalarios entre 2022 y 2024.

**Mediciones principales:**

Sensibilidad, especificidad, F1-*score* y exactitud del algoritmo basado en reglas diagnósticas y del modelo de aprendizaje supervisado.

**Resultados:**

La aplicación integra un cuestionario clínico estructurado que incluye la mayoría de los diagnósticos de cefaleas primarias recogidos en la ICHD-3. El algoritmo basado en reglas alcanzó una sensibilidad del 89,8% (IC 95%: 87,9-91,5), una especificidad del 99,4% (IC 95%: 99,2-99,5), un F1-*score* del 91,8% (IC 95%: 90,3-93,1) y una exactitud del 93,3% (IC 95%: 91,5-94,8). El modelo de aprendizaje supervisado mostró una sensibilidad del 88,1% (IC 95%: 84,0-91,8), una especificidad del 99,1% (IC 95%: 98,6-99,5), un F1-*score* del 88,5% (IC 95%: 84,6-92,0) y una exactitud del 85,5% (IC 95%: 79,3-91,4).

**Conclusiones:**

Cefalytics (https://cefalytics.com/) muestra un alto rendimiento diagnóstico y puede constituir una herramienta útil de apoyo para médicos no expertos, especialmente en atención primaria, facilitando una mayor precisión diagnóstica y una optimización terapéutica más precoz. Estos resultados requieren validación externa en una cohorte independiente en este ámbito.

## Abstract (en)

**Objective:**

To design and validate a Spanish-language artificial intelligence–based application to support the diagnosis of primary headaches by non-expert physicians.

**Design:**

Retrospective observational study for diagnostic development and validation based on clinical records.

**Setting:**

Neurology outpatient clinics receiving patients referred from primary care (level 2 headache care).

**Participants:**

A total of 1,029 adult patients with a diagnosis of 17 types of primary headache confirmed by an expert neurologist according to the criteria of the International Classification of Headache Disorders, 3 rd edition (ICHD-3), treated at two hospital centers between 2022 and 2024 were included.

**Main outcome measures:**

Sensitivity, specificity, F1-score and accuracy of the rule-based diagnostic algorithm and the supervised learning model.

**Results:**

The application integrates a structured clinical questionnaire that includes most primary headache diagnoses defined in the ICHD-3. The rule-based algorithm achieved a sensitivity of 89.8% (95% CI: 87.9-91.5), specificity of 99.4% (95% CI: 99.2-99.5), F1-score of 91.8% (95% CI: 90.3-93.1), and accuracy of 93.3% (95% CI: 91.5-94.8). The supervised learning model showed a sensitivity of 88.1% (95% CI: 84.0-91.8), specificity of 99.1% (95% CI: 98.6-99.5), F1-score of 88.5% (95% CI: 84.6-92.0), and accuracy of 85.5% (95% CI: 79.3-91.4).

**Conclusions:**

Cefalytics (https://cefalytics.com/) shows high diagnostic performance and may represent a useful support tool for non-expert physicians, particularly in primary care, facilitating greater diagnostic accuracy and earlier therapeutic optimization. These results require external validation in an independent cohort within this setting.

## Introducción

El diagnóstico de las cefaleas primarias se basa en los criterios establecidos en la tercera edición de la Clasificación Internacional de las Cefaleas de la International Headache Society (ICHD-3)^1^. Sin embargo, su aplicación en la práctica clínica puede presentar dificultades en determinadas situaciones, por ejemplo, cuando estos criterios son utilizados por profesionales con limitación de tiempo o con menor experiencia en su manejo^2^, ^3^, ^4^, lo que puede suponer una barrera en ámbitos como atención primaria^5^ o neurología general, correspondientes al nivel asistencial 2 en cefaleas^6^.

En los últimos años, el desarrollo de la inteligencia artificial (IA) en el ámbito de las cefaleas ha generado nuevas expectativas, con aplicaciones potenciales que incluyen la detección precoz de crisis de migraña, la evaluación de la respuesta al tratamiento y el apoyo al diagnóstico y clasificación de las distintas cefaleas^7^. En este último escenario, la IA puede constituir un recurso de gran utilidad clínica, tanto como herramienta de cribado como para aumentar la precisión diagnóstica en las cefaleas primarias, lo que constituye el objetivo del presente trabajo.

Partimos de la hipótesis de que una aplicación informática basada en la IA, concretamente el aprendizaje automático *(machine learning)*, puede ser una herramienta diagnóstica rápida y de fácil uso en la práctica clínica diaria. Además, permitiría una mejor aplicación de los criterios diagnósticos de la ICHD-3 en el conjunto de las cefaleas primarias y podría facilitar el diagnóstico de casos complejos, inusuales o con criterios incompletos.

Una vez desarrollada esta aplicación, nuestro objetivo es llevar a cabo su validación como paso previo a su implementación en la práctica clínica real y, en última instancia, contribuir a mejorar la atención a los pacientes con cefaleas primarias, favoreciendo un acceso más adecuado y precoz al tratamiento en función del diagnóstico establecido.

## Material y métodos

### Desarrollo de la aplicación

Mediante la colaboración entre un ingeniero de *software* y neurólogos expertos en cefaleas, se diseñó una aplicación informática en versión web (https://cefalytics.com/) concebida como herramienta diagnóstica para las cefaleas primarias. La aplicación integra 17 cefaleas primarias, correspondientes a los grupos 1 a 4 de la ICHD-3, así como la *epicránea fugax*, incluida en el apéndice^1^ (tabla 1).

**Tabla 1:** Cefaleas primarias de la ICHD-3 incluidas en la aplicación Cefalytics^a^

Grupo ICHD-3	Cefalea primaria
Grupo 1	Migraña sin aura
	Migraña con aura
	Migraña crónica
Grupo 2	Cefalea de tensión episódica
	Cefalea de tensión crónica
Grupo 3	Cefalea en racimos
	Hemicránea paroxística
	Hemicránea continua
	SUNCT/SUNA
Grupo 4	Cefalea tusígena primaria
	Cefalea por esfuerzo físico primaria
	Cefalea por actividad sexual primaria
	Cefalea punzante primaria
	Cefalea numular
	Cefalea hípnica
	Cefalea diaria persistente *de novo*
Apéndice A4	*Epicránea fugax*

ICHD-3: criterios diagnósticos establecidos en la tercera edición de la Clasificación Internacional de las Cefaleas de la International Headache Society.

a Se excluyeron la cefalea en trueno primaria, por su baja prevalencia y su posible confusión con cefaleas secundarias, así como la cefalea por estímulos fríos y la cefalea por compresión externa, por su escasa relevancia clínica.

Un neurólogo experto realizó una adaptación de los criterios diagnósticos a un formato de preguntas cerradas para su integración en la aplicación.

La aplicación incorpora un cuestionario clínico que incluye sexo, edad y 23 preguntas con respuestas dicotómicas (sí/no) o de opción múltiple (tabla 2). Las preguntas, 15 de cumplimentación obligatoria, recogen las principales características clínicas de las cefaleas primarias incluidas en la ICHD-3 y pueden completarse en menos de un minuto con la información obtenida durante la anamnesis.

**Tabla 2:** Cuestionario clínico utilizado en la aplicación Cefalytics

Variable	Opciones de respuesta
Número de episodios	< 5 / 5-9 / 10-20 / > 20
Frecuencia temporal	< 3 meses / 0-15 días-mes / > 15 días-mes / indeterminado
Duración del episodio	Segundos-minutos / minutos-horas / horas - 72 h / > 72 h
Localización	Unilateral / bilateral / cuero cabelludo
Carácter del dolor	Pulsátil / opresivo / otro
Intensidad	Leve / moderada / grave
Empeora con actividad física	Sí / No
Síntomas acompañantes	Náuseas, vómitos, fotofobia, fonofobia
Aura	Sí / No
Síntomas autonómicos	Sí / No
Inquietud o agitación	Sí / No
Trayectoria típica *(epicránea fugax)*	Sí / No
Factores desencadenantes	Tos, esfuerzo, Valsalva, actividad sexual
Despierta al paciente	Sí / No
Dolor continuo desde inicio	Sí / No
Respuesta a indometacina	Sí / No
Respuesta a triptán / ergótico	Sí / No

Un especialista en informática implementó un algoritmo computarizado basado en reglas lógicas que replica los criterios diagnósticos adaptados de la ICHD-3, permitiendo el diagnóstico automático de aquellas cefaleas que cumplen dichos criterios.

## Entrenamiento del modelo y población de estudio

Se realizó un estudio observacional retrospectivo de desarrollo y validación diagnóstica basado en registros clínicos de pacientes con diagnóstico de cefalea primaria. El reclutamiento se realizó de forma anónima entre el 1 de enero de 2022 y el 31 de diciembre de 2024 mediante un muestreo de conveniencia, llevado a cabo por 3 neurólogos expertos en cefaleas en 2 centros hospitalarios incluyendo todos los casos disponibles en el periodo de estudio. Los pacientes procedían de consultas de neurología de nivel asistencial 2 en cefaleas, remitidos desde atención primaria.

Los criterios de inclusión fueron: a) edad igual o superior a 18 años, y b) diagnóstico de cefalea primaria mediante la aplicación de los criterios de la ICHD-3 por parte de un neurólogo experto (estándar de referencia). Los criterios de exclusión fueron: a) ausencia de información clínica suficiente en la historia para completar las variables obligatorias del cuestionario, incluso en los pacientes con diagnóstico previamente establecido, y b) la presencia de síntomas o signos sugestivos de cefalea secundaria con necesidad de completar el estudio diagnóstico, según el criterio clínico del neurólogo.

De acuerdo con estos criterios, el neurólogo completó el cuestionario de la aplicación, de forma independiente al diagnóstico de referencia ya establecido previamente en función de la historia clínica, en un total de 1.053 pacientes para el entrenamiento posterior del modelo de aprendizaje automático.

## Herramientas tecnológicas y análisis estadístico

Para el desarrollo de la aplicación se empleó un conjunto de tecnologías orientadas a la inteligencia artificial que se describen en el material suplementario.

De los 1.053 casos iniciales incluidos, se excluyeron 5 correspondientes a cefaleas no contempladas en los diagnósticos seleccionados y 19 cuestionarios duplicados, definidos como aquellos con características clínicas, edad y sexo idénticos. La muestra final quedó constituida por 1.029 diagnósticos.

Las cefaleas con 11 o menos casos se agruparon en la categoría de «desconocida» para el entrenamiento del modelo, evitando el sobreajuste en clases de baja prevalencia (material suplementario). La distribución de la muestra se expone en la tabla 3.

**Tabla 3:** Distribución de casos por tipo de cefalea (n = 1.029)

Cefalea primaria	Total (n)	Entrenamiento (75%)	Prueba (25%)	Total (%)
Cefalea de tensión crónica	71	52	19	6,90
Cefalea de tensión episódica	54	40	14	5,25
Cefalea diaria persistente *de novo*	26	19	7	2,53
Cefalea en racimos	61	45	16	5,93
Cefalea punzante primaria	26	19	7	2,53
Cefalea tusígena primaria	21	14	7	2,04
Migraña con aura	124	93	31	12,05
Migraña crónica	281	209	72	27,31
Migraña sin aura	276	205	71	26,82
Cefalea numular	35	26	9	3,40
Desconocida / cefaleas poco frecuentes	54	39	15	5,25
Total	1.029	781	253	100

Para la validación interna (Python, versión 3.12.3), la cohorte se dividió en un conjunto de entrenamiento (75%) y un conjunto de prueba independiente (25%), garantizando una partición estratificada según el tipo de cefalea, de modo que se mantuviera la distribución proporcional de las distintas categorías diagnósticas en ambos subconjuntos. El conjunto de prueba permaneció completamente aislado durante el proceso de entrenamiento, y las métricas de rendimiento se calcularon exclusivamente sobre este conjunto independiente.

El rendimiento diagnóstico global se evaluó mediante el cálculo de sensibilidad, especificidad, valor predictivo positivo (VPP), valor predictivo negativo (VPN), F1-*score* macro y exactitud *(accuracy)* balanceada. Las métricas se calcularon a partir de la matriz de confusión obtenida al comparar el diagnóstico real (estándar de referencia) con el diagnóstico generado por cada algoritmo (computarizado y aprendizaje supervisado). Para cada cefalea se empleó un enfoque *one-vs-rest*. Los intervalos de confianza (IC) del 95% de sensibilidad, especificidad, VPP y VPN se estimaron mediante el método exacto binomial de Clopper-Pearson. Para el F1-*score* global y exactitud, los IC del 95% se calcularon mediante técnica *bootstrap* con 2.000 remuestreos con reemplazo sobre el conjunto de evaluación. La calibración del modelo se evaluó mediante curvas de calibración utilizando un enfoque *one-vs-rest* para cada categoría diagnóstica. Se calculó el *Brier score* como medida global de la precisión de las probabilidades predichas.

## Propiedad intelectual

La aplicación cuenta con registro de propiedad intelectual en la plataforma Safe Creative (número de registro RPISC2404047562329).

## Resultados

Tras la optimización de hiperparámetros, el modelo Gradient Boosting obtuvo el mayor F1-macro y el mejor rendimiento global en el conjunto de prueba independiente.

## Rendimiento diagnóstico global

En la muestra final de 1.029 cefaleas, el algoritmo basado en reglas proporcionó diagnóstico en 891 casos (86,6%). En 138 casos (13,4%) no se cumplieron criterios suficientes para su clasificación en un grupo específico de cefaleas primarias. El F1-*score* fue del 91,8% (IC 95%: 90,3-93,1%) y la exactitud del 93,3% (IC 95%: 91,5-94,8%) tras ponderar el análisis por la prevalencia de cada tipo de cefalea (tabla 4).

**Tabla 4:** Rendimiento diagnóstico global del algoritmo basado en reglas

Sensibilidad (IC 95%)	Especificidad (IC 95%)	VPP (IC 95%)	VPN (IC 95%)	F1-*score* (IC 95%)	Exactitud (IC 95%)
89,8% (87,9-91,5%)	99,4% (99,2-99,5%)	95,9% (95,1-96,7%)	97,9% (97,3-98,3%)	91,8% (90,3-93,1%)	93,3% (91,5-94,8%)

IC 95%: intervalo de confianza del 95%; VPN: valor predictivo negativo; VPP: valor predictivo positivo.

En el modelo de aprendizaje supervisado, el análisis ponderado mostró un F1-*score* del 88,5% (IC 95%: 84,6-92%) y una exactitud del 85,5% (IC 95%: 79,3-91,4%) (tabla 5).

**Tabla 5:** Rendimiento diagnóstico global del modelo de aprendizaje supervisado

Sensibilidad (IC95%)	Especificidad (IC 95%)	VPP (IC 95%)	VPN (IC 95%)	F1-*score* (IC 95%)	Exactitud (IC 95%)
88,1% (84,0-91,8%)	99,1% (98,6-99,5%)	90,6% (87,9-93,9%)	98,3% (97,3-99,0%)	88,5% (84,6-92,0%)	85,5% (79,3-91,4%)

IC 95%: intervalo de confianza del 95%; VPN: valor predictivo negativo; VPP: valor predictivo positivo.

## Rendimiento diagnóstico por tipo de cefalea

El rendimiento diagnóstico fue elevado en las cefaleas primarias más prevalentes, especialmente con el algoritmo basado en reglas. En la migraña, la sensibilidad osciló entre el 89,9 y el 96,8%, con especificidades próximas al 99% (99,7% en migraña crónica). En la cefalea de tensión, la sensibilidad se situó en torno al 85-87%, con especificidades igualmente elevadas. En algunas entidades menos frecuentes, la sensibilidad fue inferior, como en la cefalea numular (77,1%; IC 95%: 59,9-89,6) o en la cefalea en racimos (80,3%; IC 95%: 68,2-89,4). El modelo de aprendizaje supervisado mostró un rendimiento ligeramente menor, aunque comparable en términos globales. Los resultados completos por entidad diagnóstica se detallan en el material suplementario.

La calibración del modelo fue globalmente adecuada, con valores bajos de *Brier score* en todas las clases (rango: 0,001-0,036). Las curvas de calibración (material suplementario) mostraron una buena concordancia con la línea de identidad en las clases más frecuentes, aunque se observaron desviaciones e irregularidades en algunas clases menos representadas, relacionadas con el menor tamaño muestral.

## Diagnóstico combinado

**Figura 1:**
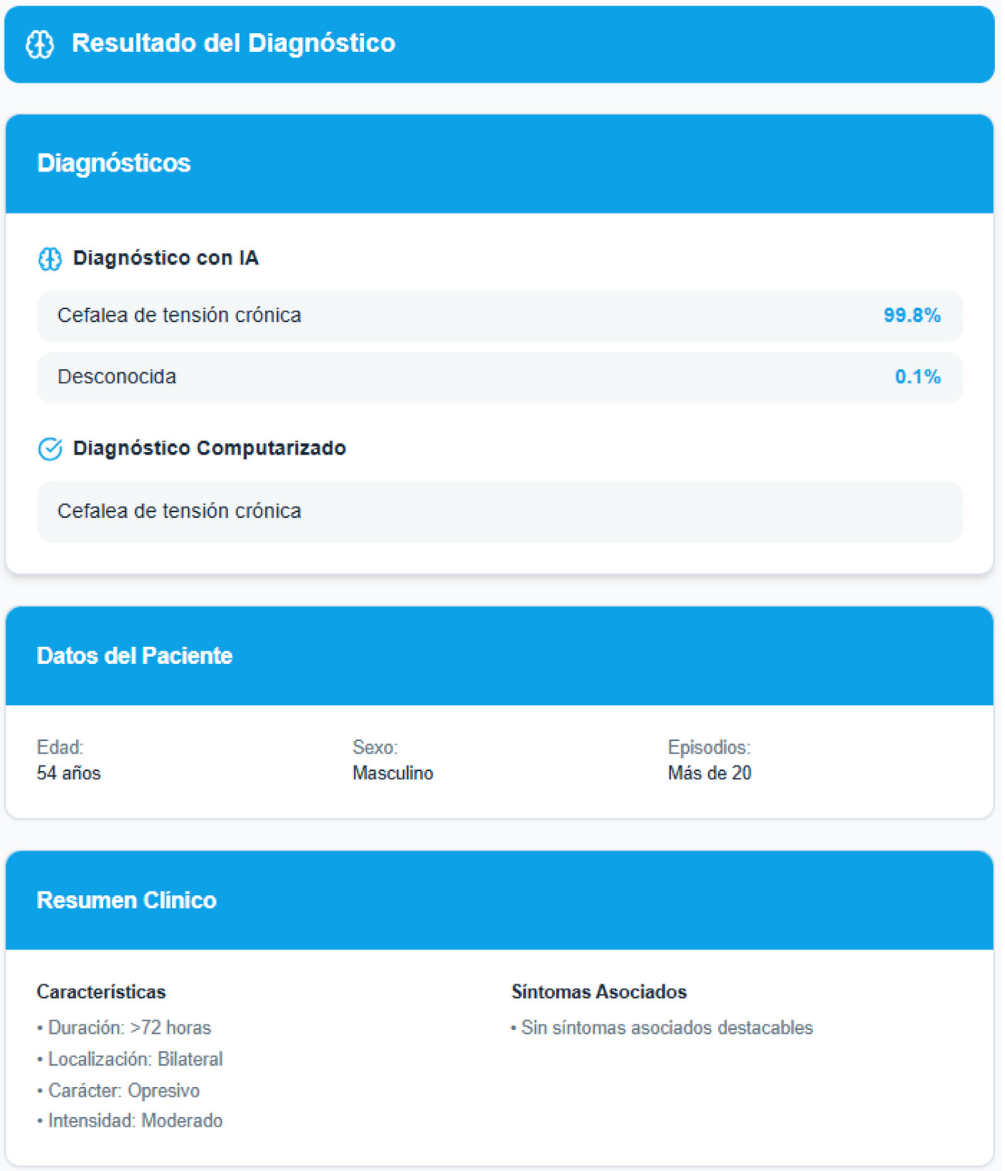
Ejemplo de diagnóstico final en Cefalytics. La aplicación muestra los 2 diagnósticos más probables del aprendizaje supervisado (IA) y, en el caso de que cumpla los criterios ICHD-3 adaptados de alguna cefalea primaria, el diagnóstico del algoritmo computarizado. IA: inteligencia artificial; ICHD-3: criterios diagnósticos establecidos en la tercera edición de la Clasificación Internacional de las Cefaleas de la International Headache Society.

La aplicación integra un algoritmo basado en reglas y un modelo de aprendizaje supervisado. Esta aproximación híbrida permite complementar ambos métodos cuando no se cumplen estrictamente los criterios de la ICHD-3 o en cefaleas poco prevalentes con menor rendimiento del modelo supervisado (fig. 1).

## Discusión

En este estudio se presenta el desarrollo y la validación de una aplicación informática en castellano destinada a apoyar el diagnóstico de las cefaleas primarias por médicos no expertos, fundamentalmente en atención primaria y en neurología general. La aplicación se basa en los criterios de la ICHD-3^1^ e integra un sistema híbrido que combina un algoritmo computarizado basado en reglas con un modelo de aprendizaje supervisado, entrenado a partir de una amplia cohorte clínica real.

Los resultados obtenidos muestran un rendimiento diagnóstico elevado, especialmente para las cefaleas primarias más prevalentes, como la migraña sin aura, la migraña con aura y la migraña crónica. En el modelo de aprendizaje supervisado, el F1-*score* global fue del 88,5% valor que se sitúa en un rango comparable o superior al descrito en estudios previos^8^. Por su parte, el algoritmo computarizado basado en reglas alcanzó un F1-*score* del 91,8%, aunque su utilidad se limita estrictamente a los casos que cumplen los criterios diagnósticos adaptados de la ICHD-3.

La aplicación de la inteligencia artificial al ámbito de las cefaleas ha experimentado un notable crecimiento en los últimos años. En una revisión sistemática reciente se identificaron numerosos trabajos centrados en el uso de técnicas de aprendizaje automático para el diagnóstico de cefaleas; si bien ninguna estaba disponible en castellano^8^.

Las principales aplicaciones basadas en cuestionarios utilizan entre 17 y 75 preguntas^8^, ^9^, ^10^ y, en general, se han centrado en un número más limitado de diagnósticos. Así, en el estudio de Simic et al.^9^ el diagnóstico final se restringió a migraña, cefalea de tensión u otras cefaleas, mientras que en el de Kwon et al.^10^ se incluyeron migraña, cefalea tensional, cefaleas epicraneales, cefaleas trigémino-autonómicas y cefalea en trueno (única cefalea secundaria). En esta última la sensibilidad y especificidad fueron del 65 y 51%^10^, respectivamente, lo que pone de manifiesto las limitaciones de estos enfoques en las cefaleas secundarias.

En nuestro caso, la herramienta se ha centrado exclusivamente en las cefaleas primarias, dado que la identificación de banderas rojas de cefaleas secundarias suele requerir datos clínicos adicionales, incluidos hallazgos de la exploración, que difícilmente pueden recogerse mediante un cuestionario breve^11^, ^12^.

En cuanto al rendimiento diagnóstico, Simic et al.^9^ reportaron un F1-*score* en torno al 75% para sus 3 categorías diagnósticas, mientras que Kwon et al.^10^ obtuvieron una exactitud del 81%. El estudio más relevante corresponde a un grupo japonés^13^, ^14^, ^15^, que incluyó 6.058 participantes clasificados en 5 grupos diagnósticos y utilizó un cuestionario de 17-22 preguntas, alcanzando un F1-*score* del 84% y mejorando la concordancia entre médicos expertos y no expertos en cefalea. Este grupo es además el único que ha realizado una validación externa, aunque en una muestra limitada (n = 59), obteniendo un F1-*score* del 98% para migraña^16^. De forma consistente, en los estudios revisados^8^, el rendimiento fue inferior en las cefaleas de baja prevalencia.

Un aspecto diferencial de nuestra aplicación es la combinación de un modelo de aprendizaje supervisado con un algoritmo basado en reglas diagnósticas. Estas herramientas computarizadas «pre-ML» se han desarrollado para intentar mejorar la precisión diagnóstica de las cefaleas. En una revisión sistemática^17^, se observó que el 50% son autoadministradas por el paciente y buscaban objetivos similares a los nuestros^17^, ^18^, ^19^, ^20^, ^21^, ^22^, ^23^, ^24^. Sin embargo, solo una de ellas estaba disponible en castellano (fines epidemiológicos)^22^. Recientemente se ha publicado una aplicación móvil en castellano, basada en reglas, para ayudar al diagnóstico de migraña en atención primaria que, no obstante, presenta importantes diferencias con la nuestra, ya que es autoadministrada por el paciente y solo permite el diagnóstico de 3 entidades, migraña, cefalea de tensión o cefalea mixta, esta última no reconocida por la ICHD-3^25^.

En nuestra aplicación, este sistema híbrido, también utilizado por otros autores^9^, ^24^, permite complementar las limitaciones de cada enfoque: el modelo de aprendizaje automático proporciona una estimación probabilística útil en las cefaleas más frecuentes, mientras que el algoritmo basado en reglas facilita el diagnóstico de cefaleas primarias cuando se cumplen estrictamente los criterios diagnósticos adaptados de la ICHD-3.

Nuestro estudio presenta claras limitaciones. En primer lugar, la ausencia de validación externa^16^ fuera de nuestros 2 centros y especialmente en un ámbito distinto como la atención primaria, limita la generalización de los resultados y su aplicabilidad clínica.

En segundo lugar, existe riesgo de sesgos de información y de selección que podrían haber sobreestimado el rendimiento observado, ya que el diagnóstico de referencia y la cumplimentación del cuestionario fueron realizados por el mismo especialista, y la muestra se reclutó mediante muestreo de conveniencia. En definitiva, nuestra herramienta requiere otros estudios para confirmar su validez y aplicabilidad clínica. Por otro lado, no permite el diagnóstico de cefaleas secundarias^14^, ^26^ y está dirigida exclusivamente a los pacientes adultos^27^.

Entre las fortalezas del estudio destacan la inclusión de casi todas las cefaleas primarias recogidas en la ICHD-3, el uso de una cohorte clínica amplia y bien caracterizada, el desarrollo de la aplicación en castellano y su adaptación al entorno asistencial real. Asimismo, el diseño metodológico cumple los principales criterios recomendados por la guía TRIPOD-AI para estudios clínicos que utilizan aprendizaje supervisado^19^, ^28^, incluyendo una pregunta de investigación claramente definida, una fuente de datos bien descrita, un estándar de referencia clínico claro, aunque siempre susceptible de la variabilidad interobservador entre investigadores, la evaluación del rendimiento en una muestra independiente de la utilizada para el entrenamiento del modelo, la calibración del modelo e incluir sus limitaciones, entre las que destacan la ausencia de validez externa para su aplicación clínica y el alto riesgo de sesgos.

La adherencia a las guías metodológicas sobre la aplicación de la IA, PROBAST-AI o TRIPOD-AI^28^, en cefaleas es un tema crucial en la actualidad^29^ y necesario para conocer la metodología adecuada y las limitaciones existentes en los trabajos previos^8^.

En conclusión, se ha desarrollado y validado una aplicación informática en castellano, Cefalytics, de uso rápido y sencillo, para el apoyo al diagnóstico de las cefaleas primarias en atención primaria y neurología general. La aplicación combina un algoritmo computarizado basado en los criterios de la ICHD-3 con un modelo de aprendizaje supervisado entrenado en una cohorte clínica amplia, lo que permite un rendimiento diagnóstico elevado, especialmente en las cefaleas más prevalentes. No obstante, nuestros resultados requieren validación externa en una cohorte independiente de atención primaria antes de su aplicación clínica en este ámbito, caracterizado por alta carga asistencial y variabilidad en la formación específica en cefaleas. Cefalytics podría integrarse como una herramienta de apoyo estructurado a la práctica clínica, facilitando una mayor precisión diagnóstica^30^ en cefaleas primarias con su doble diagnóstico final en muchos casos, y contribuyendo de este modo a una optimización terapéutica. Por otro lado, permitiría una agilización del tiempo de consulta evitando demoras en la aplicación de los criterios de la ICHD-3, especialmente en las cefaleas menos frecuentes.

**Lo conocido sobre el tema**

• El diagnóstico de las cefaleas primarias se basa en los criterios de la Clasificación Internacional de Cefaleas (ICHD-3), cuya aplicación en la práctica clínica presenta dificultades en determinadas entidades y ámbitos como la atención no especializada.
• La inteligencia artificial y el aprendizaje automático se han utilizado como herramientas de apoyo diagnóstico en cefaleas, principalmente en migraña, aunque con aplicaciones limitadas en lengua española.

**Qué aporta este estudio**

• Se ha desarrollado y validado una aplicación informática en castellano que facilita el diagnóstico de las cefaleas primarias mediante un cuestionario breve y de rápida administración.
• La aplicación combina un algoritmo computarizado basado en los criterios ICHD-3 con un modelo de aprendizaje supervisado entrenado en una cohorte clínica amplia.
• Esta herramienta puede ayudar a médicos no expertos en cefaleas, especialmente en atención primaria y neurología general, a mejorar la precisión diagnóstica y la adecuación terapéutica, aunque precisa validación externa en una cohorte independiente.

## Autoría

Todos los autores han contribuido sustancialmente al diseño del estudio, análisis e interpretación de los datos, redacción o revisión crítica del manuscrito y aprobación de la versión final para su publicación. Todos los autores asumen la responsabilidad del contenido del trabajo y cumplen los criterios de autoría establecidos por el ICMJE.

## Financiación

No se ha recibido financiación para la realización de este artículo.

## Consideraciones éticas

El estudio se llevó a cabo de acuerdo con los principios éticos para la investigación médica en seres humanos establecidos en la Declaración de Helsinki de la Asociación Médica Mundial y con los Requisitos de uniformidad para manuscritos enviados a revistas biomédicas. El protocolo fue evaluado y aprobado por el Comité de Ética de la Investigación Provincial de Almería (código 25/2022). Se garantizó en todo momento la confidencialidad de la información y la protección de datos personales conforme a la normativa vigente en materia de protección de datos, asegurando el respeto a los derechos de privacidad de los participantes.

## Consentimiento informado

El Comité de Ética de la Investigación Provincial de Almería (código 25/2022) eximió de la obtención de consentimiento informado debido al carácter retrospectivo y anonimizado del estudio, basado en registros clínicos previamente existentes. No se incluyeron datos identificativos y se garantizó la confidencialidad conforme a la normativa vigente.

## Uso de IA

En la elaboración del manuscrito no se ha utilizado inteligencia artificial generativa para la redacción del contenido científico. Las herramientas de inteligencia artificial empleadas en el estudio forman parte del objeto de investigación (desarrollo y validación de la aplicación Cefalytics) y se describen detalladamente en la sección de Material y Métodos.

## Conflicto de intereses

Los autores declaran no tener conflicto de intereses.
